# Low persistence of a monocarpic invasive plant in historical sites biases our perception of its actual distribution

**DOI:** 10.1111/j.1365-2699.2011.02677.x

**Published:** 2012-01-24

**Authors:** Jan Pergl, Petr Pyšek, Irena Perglová, Vojtěch Jarošík, Şerban Procheş

**Affiliations:** 1 Department of Invasion Ecology, Institute of Botany, Academy of Sciences of the Czech RepublicCZ-252 43 Průhonice, Czech Republic; 2 Department of Ecology, Faculty of Science, Charles University PragueViničná 7, CZ-128 44 Prague, Czech Republic

**Keywords:** Biological invasions, Czech Republic, *Heracleum mantegazzianum*, herbarium records, invasive species, land use, species distribution, species persistence

## Abstract

**Aim:**

As accurate and up-to-date distribution data for plant species are rarely available, cumulative records over long periods of time are frequently used for mapping distributions, without taking into account that species do not persist in their historical localities forever. However, persistence is highly relevant in changing modern landscapes, especially for invasive species that dynamically spread in unstable human-made habitats. We studied how an invasive species, *Heracleum mantegazzianum*, persists at sites once colonized and how its ability to persist affects its distribution.

**Location:**

The Czech Republic.

**Methods:**

We visited 521 localities of *H. mantegazzianum* occurrence reported in the literature and herbaria to determine whether the species still occurs at these sites. By using *G*-tests and classification trees, we explored the roles of various factors affecting its persistence at a site.

**Results:**

Of the total number of 521 historical sites at which the species has occurred since the end of the 19th century, it persists at only 124 (23.8%). The persistence rate differs with respect to habitat type and is highest in meadows and forest margins. Analysis using classification trees indicated that the factors that best explain persistence are: type of habitat (with meadow and forest margins over-represented); urbanity (with a higher persistence outside urban areas); proximity to the place of the species’ introduction into the country; metapopulation connectivity; and distance to the nearest neighbouring population.

**Main conclusions:**

The use of cumulative historical records as a measure of species distribution, which is common in invasion literature, can seriously overestimate the actual distribution of alien plant species with low persistence. In the case of alien species such as *H. mantegazzanium*, which is non-clonal and reproduces only by seed, estimates of distribution and spread based on historical data are informative about potentially suitable habitat but may be unreliable as indicators of current occurrence and invasion dynamics.

## Introduction

Invasion ecologists have long been interested in studying the colonization ability of alien species (Mack, 2000; Davis, 2009). Common methods range from direct observations made at small scales over short periods of time to the reconstruction of species distributions and spread over long periods using literature and herbarium records (Pyšek, 1991; Pyšek & Prach, 1993; Delisle *et al.*, 2003; Aikio *et al.*, 2010a). At small scales, the spatial and temporal persistence of species, namely their ability to survive at a site once it is occupied, has been intensively studied for both alien and native species (e.g. Law & Morton, 1996; Stöcklin & Fischer, 1999; Chesson, 2000; Tilman, 2004). At the landscape level, species persistence is affected by several factors, including the distribution of suitable habitats and their characteristics, and the metapopulation dynamics of species. The most important habitat characteristics include resource availability, disturbance regime, and habitat dynamics over time, which in modern landscapes reflect changes in land use (Davis *et al.*, 2000; Vilà & Gimeno, 2001; Chytrý*et al.*, 2005, 2008a,b).

Data on the persistence of species at large spatial scales and over longer periods of time are much more common for native species (Tingley & Beissinger, 2009) and have been used to address conservation issues, for example to evaluate the effectiveness of protection measures (Primack *et al.*, 2009) and to assess long-term changes in species diversity (Pyke & Ehrlich, 2010). This applies mainly to small isolated populations of native species that are threatened by stochastic environmental and demographic processes or decreased fitness (Brassil, 2001; Dennis, 2002). However, there are only a few robust data sets that document how long these species persist in sites once they are colonized (e.g. for *Swertia perenis*, Lienert *et al.*, 2002; for *Dryopteris cristata*, Landergott *et al.*, 2000; and a multispecies study by Kery *et al.*, 2006). For alien species that typically spread quickly and undergo large population fluctuations in space and time (Pyšek & Hulme, 2005), such data are even rarer, despite their utility for revealing the dynamics of plant invasions on a historical time-scale. To our knowledge, only two studies have addressed the historical persistence of alien species at a regional spatial scale: a study on three invasive clonal species in the Czech Republic (*Fallopia japonica*, *F. sachalinensis* and *Rudbeckia laciniata*; Pyšek *et al.*, 2001) and a brief report on the persistence of the monocarpic perennial *Heracleum mantegazzianum* in Ireland (Wade *et al.*, 1997). The latter study, however, was based on limited data resulting from a public inquiry and merely reported numbers of surviving populations.

Our understanding of the factors that shape the realized distribution of alien species following their invasion of a new range is limited and therefore represents a source of potential bias, especially when distribution is inferred from species records accumulated over time. Although methods for correcting non-detected occurrences exist (MacKenzie *et al.*, 2002; Kery *et al.*, 2006), they are rarely used. Generally, the lack of long-term data on population trends of invasive species (e.g. Müllerová*et al.*, 2005) is compensated for by using correlative approaches based on reported species occurrences. Various approaches have been employed to account for missing records and variation in sampling intensity over time (Delisle *et al.*, 2003; Wu *et al.*, 2005; Aikio *et al.*, 2010b).

Nevertheless, the potential to overestimate species distributions when considering all known records of occurrence (without accounting for persistence) is rarely acknowledged and has never been tested using rigorous data. What is largely neglected is that the mapped distribution may be smaller than reported or may differ from reality owing to local attrition and colonization. This is even more pronounced in the case of alien species, where most attention is paid to spread and dispersal processes over large distances (see Pyšek & Hulme, 2005 and references therein) or to colonization at the scale of individual habitats (Chytrý*et al.*, 2008b). The distribution is also likely to be overestimated in species subjected to current or past control campaigns, and in species with fast spread and high population turnover such as short-lived annuals. In contrast, the persistence of clonal invasive species on a time-scale of decades to centuries is rather high (Pyšek *et al.*, 2001; Lienert *et al.*, 2002).

In this study, we explore the persistence of a monocarpic invasive species, *Heracleum mantegazzianum*, in sites and habitats that the species once invaded, at a spatial scale that includes the entire Czech Republic (78,000 km^2^), and at a historical temporal scale of up to 120 years. Detailed data on the invasion history of this species in the Czech Republic are available. However, it is unclear how population dynamics, human-mediated and natural long-distance dispersal events, and recent eradication efforts have affected its distribution over time. We ask the following questions. (1) How does the species persist in sites once colonized? (2) What are the ecological and human-related factors that determine whether the species persists in a site or not? (3) What is the actual current distribution of *H. mantegazzianum* and how does it differ from the distribution that can be derived from the historical literature? Finally, if it is different, (4) what implications could such bias have on our perception of the ecology, distribution and historical invasion dynamics of alien species?

## Materials and methods

### Study species

*Heracleum mantegazzianum* Sommier & Levier (Apiaceae) is a monocarpic, short-lived perennial hemicryptophyte that reproduces exclusively by seed (Krinke *et al.*, 2005; Moravcová*et al.*, 2006; Pergl *et al.*, 2006). The species is able to self-pollinate, can produce up to 50,000 seeds per plant (Perglová*et al.*, 2006), and has good dispersal ability (Moravcová*et al.*, 2010; Pergl *et al.*, 2011). Its native distribution is in the Western Greater Caucasus, where it grows in tall-herb meadows, forest clearings and along forest margins below the tree line (Ochsmann, 1996). In 1817 it was introduced to Europe as a garden ornamental and it is now considered as invasive or naturalized in many European countries, central Russia and North America (Page *et al.*, 2006; Pyšek *et al.*, 2008; DAISIE, 2009).

The species is harmful to humans on account of its phytotoxic sap (Nielsen *et al.*, 2005). Its exotic appearance and enormous size (height of flowering plants up to 4.5 m), together with its direct effect on human health, made it popular among botanists, leading to a thorough knowledge of its biology and ecology (Ochsmann, 1996; Tiley *et al.*, 1996; Page *et al.*, 2006; Pyšek *et al.*, 2007a), and to detailed historical information on its distribution in some countries (Pyšek & Pyšek, 1995; Pyšek *et al.*, 2007b, 2008). It is known to have a negative effect on invaded plant communities, resulting in reduced native species diversity (Hejda *et al.*, 2009; Thiele *et al.*, 2010).

## Data

The study was carried out in the Czech Republic (Central Europe). The historical distribution was taken from the list of records published in Pyšek & Pyšek (1994); this list was updated up to the year 2007 with additional records from local herbaria, recent literature and unpublished data. From the resulting list of *c*. 650 localities, we selected sites for which the original source contained sufficient information to locate the site (in some cases supplemented by interviews with local people). Sites from the highly invaded region in western Bohemia, where the species was originally introduced to the Czech Republic (Pyšek, 1991; Müllerová*et al.*, 2005), were excluded because it was not possible to distinguish among individual populations. The individual localities were considered as separate sites when they were at least 0.5 km apart.

**Figure 1:**
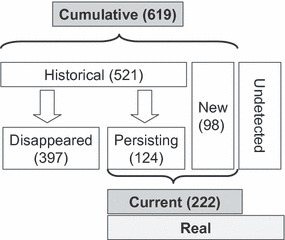
Types of sites identified in this study. ‘Historical’ sites are those where the occurrence of *Heracleum mantegazzianum* was reported up to 2007. The species still occurs at some of these sites (‘Persisting’), whereas it has disappeared from others, as inferred from its absence during systematic revisiting in the period 2008–2009 (‘Disappeared’). This recent research yielded information on new sites in which the species had not been recorded in the past (‘New’). ‘Current’ sites therefore represent the actual distribution confirmed by the recent survey, while ‘Cumulative’ distribution refers to all sites, both ‘Historical’ and ‘New’, from which the species has ever been reported. Finally, the ‘Real’ distribution takes into account that there are ‘Undetected’ sites that escaped recording both in historical data up to 2007, and in the recent sampling in 2008–2009. The numbers in brackets show the representation of individual sites for *H. mantegazzianum*.

This search yielded 521 sites that were invaded by *H. mantegazzianum* at some point between 1877, which is the date of the first record of the species in the wild in the Czech Republic (Holub, 1997), and 2007 (referred to hereafter as ‘historical sites’; Fig. 1). These sites were visited in 2008 and 2009 to verify the presence of the species; the visits were made during the species’ flowering time (June–July). If new sites with a *H. mantegazzianum* population (i.e. those that could not be assigned to any of the historical sites) (Fig. 1) were found when visiting historical sites, they were also recorded; we searched for them within a radius of *c*. 1–2 km of the historical site. As the revisited historical sites were distributed throughout the country, the occurrences recorded during the sampling of these sites reflect the current distribution reasonably well. Distinguishing the sites in this way made it possible to compare the distributions of *H. mantegazzianum* in the study region based on cumulative and on current data, using the common grid system for mapping species distributions in Central Europe (6′ latitude × 10′ longitude; Williamson *et al.*, 2005).

Geographical coordinates were obtained for each revised or newly recorded site using GPS, and the following characteristics were recorded in the field.

1. Habitat, grouped as follows: *riparian* (banks of rivers, brooks and ponds); vicinity of *roads*; vicinity of *paths*; *railway* embankments and their vicinity; *industrial* sites in cities; public *parks*, including green areas and private gardens in cities and villages; *orchards*; disturbed *ruderal* sites in the landscape such as rubbish tips or quarries; managed and unmanaged *meadows*; *arable fields*; *forest margins* including shrubby edges. Some sites were assigned to more than one habitat type.
2. Intensity of human impact: each site was classified according to its location with respect to the limits of the nearest human settlement; this characteristic is termed ‘*urbanity*’ (yes, sites inside; no, outside the city/village; with sites occurring at the edge of the settlement classified in both categories).
3. The earliest year of record of *H. mantegazzianum* at a given historical site was used as a measure of the residence time at the site (*year*).
4. Climatic characteristics (*mean January* and *June temperature, sum of annual precipitation*) were obtained from a climate atlas (Quitt, 1971). Other characteristics, describing the possible effects of metapopulation dynamics, were assigned to each site using ArcGIS 9.2 (http://www.esri.com).
5. A buffer zone with a perimeter of 10 km was delimited around each site, and the total length of *roads* and *rivers*, as a surrogate of connectivity, was recorded. The number of other historical and new sites of *H. mantegazzianum* in the buffer zone was used as a measure of *metapopulation connectivity* and regional infestation.
6. The connectivity with potential source populations at the regional scale was characterized by using the following parameters: distance from the nearest neighbouring site (*nearest-neighbour distance*) and distance from the place of the first introduction into the Czech Republic (Lázně Kynžvart; *distance to origin*).
7. A map of landscape protected areas and national parks (CENIA, http://www.cenia.cz) was used to determine whether the site was located within a nature protection area (*protected areas*).

### Statistical analysis

To test whether the persistence of the species at historical sites was lower or higher than expected by chance, counts of occurrences in the individual habitats, inside and outside the settlements, and inside and outside the protected areas, were compared by *G*-tests on contingency tables (e.g. Sokal & Rohlf, 1995). We used the same test to assess whether the observed distribution of records at historical and new sites in the individual habitats differs from that expected by chance. To ascertain in which habitats the records appeared lower or higher than could be expected by chance, adjusted standardized residuals of *G*-tests were compared with critical values of the normal distribution following Řehák & Řeháková (1986).

To test the roles of individual habitat types, climatic variables, geography, and human pressure on the persistence of populations at historical sites, classification trees (Breiman *et al.*, 1984; De'ath & Fabricius, 2000) were used. The trees were constructed using binary recursive partitioning, with the default Gini index impurity measure used as the splitting index, in cart 6.0 (Breiman *et al.*, 1984; Steinberg & Colla, 1995). To find an optimal tree, a sequence of nested trees of decreasing size, each being the best of all trees of its size, was produced, and their re-substitution relative errors, corresponding to residual sums of squares, were estimated. Ten-fold cross-validation was used to obtain estimates of cross-validated relative errors for these trees. Following De'ath & Fabricius (2000), a series of 50 cross-validations was run, and the most likely (modal) single minimum-cost tree was chosen for description.

## Results

### Pattern of persistence of *Heracleum mantegazzianum* in historical sites and habitats

Historically, *H. mantegazzianum* was most frequently reported from forest margins (26.7% of the total number of records), riparian habitats (21.5%), roadsides (20.9%) and meadows (20.5%), with other habitats markedly less represented (Table 1). Of the total number of 521 historical sites visited at which *H. mantegazzianum* was recorded between 1899 and 2006, the species persisted in 124 sites, resulting in a total persistence of 23.8% across habitats. The persistence across individual habitat types was non-randomly distributed (*G* = 71.93; d.f. = 10; *P* < 0.001) and ranged from 11.5% to 54.2%, with *H. mantegazzianum* in meadows and forest margins persisting significantly (*P* < 0.05) more than expected based on the pooled data, and in roads and parks significantly less; the persistence in other habitats types did not differ from the expected value (Table 1). Persistence differed significantly with respect to urbanity (*G* = 37.47; d.f. = 1; *P* < 0.001), with a higher proportion of persisting populations outside (34.2%) than inside (12.8%) urban areas. Of the total number, 188 sites were located inside protected areas (landscape protected areas, national parks, small-scale nature reserves), with persistence significantly (*G* = 21.21; d.f. = 1; *P* < 0.001) higher outside (30.0%) than inside (12.8%) protected areas.

**Table 1:** Populations of *Heracleum mantegazzianum* in the Czech Republic persisting in revisited historical sites, and those recorded at new sites during the study. Numbers of populations are divided according to habitat, and historical sites are further separated into sites from which the species disappeared and those in which the species still occurs. Habitats are ranked according to *H. mantegazzianum* percentage persistence in the historical sites. Higher and lower values for disappearance and persistence than expected by chance (values in brackets) are marked by asterisks (*< 0.05; **< 0.01; ***< 0.001). Note that a site can be assigned to more than one habitat type. Terms used for the column headings correspond to the scheme in Fig. 1 and are described in Materials and Methods

	Historical sites				New sites
		
Habitat	Persistence (%)	Total	Disappeared	Persisting	
Meadow	54.2	107	49 (72.9)**	58 (34.1)***	47
Arable field	52.4	21	10 (14.3)	11 (6.7)	12
Forest margin	43.2	139	79 (94.6)	60 (44.3)*	24
Riparian	31.3	112	77 (76.3)	35 (35.7)	23
Path	29.7	64	45 (43.6)	19 (20.4)	13
Ruderal	29.6	27	19 (18.4)	8 (8.6)	4
Industrial	27.3	11	8 (7.5)	3 (3.5)	1
Railway	22.2	36	28 (24.5)	8 (11.5)	0
Orchard	16.2	37	31 (25.2)	6 (11.8)	5
Road	14.7	109	93 (74.2)*	16 (34.8)***	24
Park	11.5	61	54 (41.5)	7 (19.5)***	2

The 98 new sites of *H. mantegazzianum* recorded during our sampling were distributed among habitats in a pattern similar to that of historical localities (Table 1); most new localities came from meadows (28.1%), forest margins (21.6%), road margins (14.4%) and riparian habitats (13.0%). The majority (79.6%) of newly recorded sites were from outside settlements.

**Figure 2:**
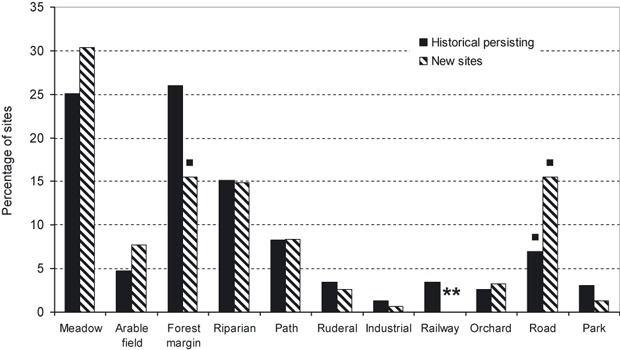
Frequency distribution of habitats in historical sites of *Heracleum mantegazzianum* (those in which the species persisted) and in new sites in the Czech Republic. The overall distribution among persisting and new sites is non-random (*G* = 23.85; d.f. = 10; *P* < 0.01), with habitats having marginally (*P* < 0.1) fewer or more records than expected by chance marked by dots, and the habitat with highly significantly (*P* < 0.01) fewer records than expected by chance in new sites marked by two asterisks.

Moreover, these data allowed us to compare the frequency distribution of habitats in which the species has been persisting with newly invaded habitats (Fig. 2). This comparison revealed a rather stable turnover in some habitats such as riparian sites, in orchards, along paths in the open landscape and in ruderal habitats, but the dynamic nature of spread was reflected by a high proportion of new localities along roads (where persistence is low). In contrast, the current populations in forest margins, and even more so in railway embankments and their close vicinity, were recruited mostly from historical populations and did not represent independent *de novo* invasion events (Fig. 2).

### Factors determining the persistence in historical localities

**Figure 3:**
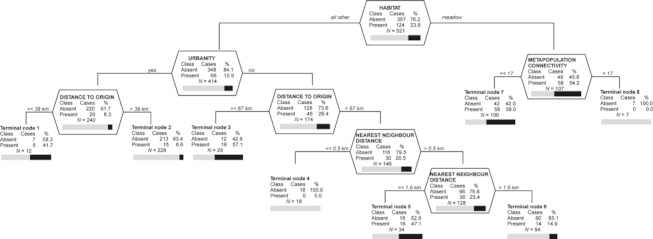
Classification tree for the persistence of *Heracleum mantegazzianum* at the investigated historical sites in the Czech Republic. Each splitting node (polygon with splitting variable name and splitting criterion) and each terminal node shows the number (*N*) of persisting (present) and non-persisting (absent) populations, expressed by numbers (cases and percentage) and horizontal bars (present in black; absent in grey).

The best classification tree (Fig. 3) had eight terminal nodes and an overall misclassification rate of 20.7%. Misclassification of non-persisting populations of *H. mantegazzianum* was 19.9%, and misclassification of persisting populations was 23.4%. The factors that best explained the persistence were habitat, urbanity, distance to origin, metapopulation connectivity and nearest-neighbour distance. Populations in meadows exhibited higher persistence than those in all other habitat types. If the meadow site had low metapopulation connectivity, that is, if the number of populations in its surrounding buffer zone was lower than 17, the population persisted with higher probability than did populations with metapopulation connectivity above this threshold.

Populations in habitat types other than meadows exhibited lower persistence if they were located in urban areas and were more than 39 km from the origin, that is, the region where the species was first introduced to the country. Those located outside urban areas persisted better than did those in settlements, and their persistence also depended on the distance to origin, with a threshold of 67 km. Those located more than 67 km from the origin were differentiated, in terms of persistence, with respect to the nearest-neighbour distance, with those located between 0.5 and 1.6 km from the next population being most likely to persist.

### Distribution inferred from data on persisting populations

**Figure 4:**
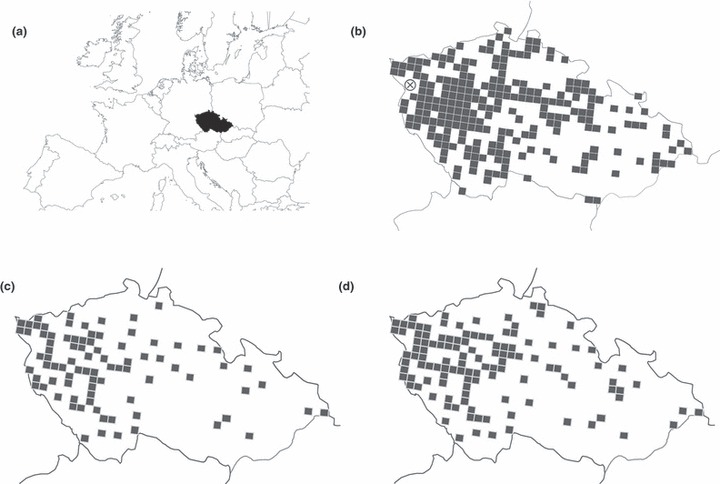
(a) Overview map showing the location of the study area in the Czech Republic. The remaining panels show grid maps for (b) all historical records of *Heracleum mantegazzianum* revised by this study in a 6′ latitude × 10′ longitude mapping system; (c) the confirmed persistence of *H. mantegazzianum* at historical sites; and (d) the current distribution within the Czech Republic combining the persisting sites and newly recorded localities (2008–2009). Localities in the west Bohemian neighbourhood of Lázně Kynžvart [see Materials and Methods; marked ⊗ in panel (b)] are excluded.

The data made it possible to compare the distributions of *H. mantegazzianum* in the study region based on historical and actual data (Fig. 4). The map based on cumulative historical distribution up to 2007 suggested that 237 of the 672 grid cells in the Czech Republic were occupied by *H. mantegazzianum*, namely 35.2% (Fig. 4a). In fact, the species is now present in only 86 of the previously recorded grid cells (12.8% of the total number of grid cells in the country) (Fig. 4b). Therefore, a more realistic insight into its distribution can be gained by summing the grid cells in which it persisted with those from which it was newly recorded, giving a total number of 120; i.e. 17.8% of grid cells in the Czech Republic currently occupied (Fig. 4c). Cumulative data overestimate the actual distribution of *H. mantegazzianum*.

## Discussion

### Historical persistence of *Heracleum mantegazzianum* compared with other invasive species

Our data indicate that *H. mantegazzianum* disappeared from 76% of the total number of sites once colonized between the end of the 19th century and the present. The persistence rate in the Czech Republic is therefore markedly lower than that of 45% reported from Ireland for the same species (Wade *et al.*, 1997). Apart from geographical differences in climate and land use between the two countries, as well as differences in the size and quality of the data sets (the Irish study was based on a much smaller data set of 96 sites, compiled by interviewing stakeholders, and lacked an analysis of site characteristics and other factors relating to persistence), a possible explanation for the different findings is increased interest in the management and control of alien species. The Irish screening was completed by 1993. In the last few decades, *H. mantegazzianum* has been the subject of media coverage, leading to increased public awareness of the problem in the study region, and resulting in a number of local control campaigns (Nielsen *et al.*, 2005; Pyšek *et al.*, 2007c). These campaigns seem to have been particularly effective in protected areas targeted by conservation authorities, where the persistence was half (12.7%) of the average figure. The obvious role of management efforts is supported by comparing these results with data on a rare native species, *Swertia perennis*, which is not subject to control efforts, and which has much higher persistence rates. Revisiting sites identified from old herbarium records revealed that this long-lived perennial species persisted in 76% of sites, disappearing from only 24% as a result of change of land use (Lienert *et al.*, 2002).

The marked difference between the persistence of *H. mantegazzianum* and that reported previously for other invasive species (Pyšek *et al.*, 2001) can be explained by life-form. Such comparison is valid in the case of, for example, *Fallopia* and *Rudbeckia* taxa, which are the subject of comparable management efforts. *Heracleum mantegazzianum* is a short-lived monocarpic perennial (Pergl *et al.*, 2006) that reproduces exclusively by seed and forms a short-term-persistent seed bank (Moravcová*et al.*, 2006). This reproductive strategy makes *H. mantegazzianum* populations dependent on habitat disturbance and on the existence of microsites that favour its germination, early growth and subsequent completion of the life cycle. This may impose a considerable disadvantage on *H. mantegazzianum* compared with clonal invasive species that have been subject to similar eradication efforts in similar habitats and yet exhibit much higher persistence rates (exceeding 70%; Pyšek *et al.*, 2001). This conclusion is supported by studies addressing native species that show that those with long-term-persistent seed banks have lower extinction rates than those with short-lived seed banks (Stöcklin & Fischer, 1999). In another study comparing trends in native species abundances over time, therophytes and geophytes exhibited much higher extinction rates than did other life-forms (Stehlik *et al.*, 2007).

### Differences in persistence among habitats

Data on native species suggest that habitat destruction or change in land use are the most significant drivers of plant species loss and their diminishing ability to persist in a region (Lienert *et al.*, 2002; Stehlik *et al.*, 2007; Primack *et al.*, 2009). However, the persistence of individual species in specific habitat types differs. Pyšek *et al.* (2001) found significant differences between the persistence of three clonal species (*Fallopia japonica*, *F. sachalinensis* and *Rudbeckia laciniata*) among the main groups of habitat types, with the highest persistence in the transport network and riparian habitats, and inter-specific differences reflecting the colonization potential and competitive abilities of the species studied. *Heracleum mantegazzianum* shows a similar pattern of persistence to *R. laciniata*, with some differences that can be attributed to eradication efforts targeted at *H. mantegazzianum*. The most striking differences in the persistence of this species, namely between sites located inside and outside urban areas, can be attributed to increasing public awareness, resulting in the removal of the species from human settlements and their neighbourhood. For example, the low persistence found in road verges, parks and similar habitats reflects increased eradication efforts at sites located close to human settlements, where the species is highly visible and attracts the attention of stakeholders. In contrast, the high persistence in meadows and forest margins indicates the importance of less disturbed habitats with lower human pressure under which the species maintains populations over a long time.

### Factors determining the historical persistence of *Heracleum mantegazzianum*

Habitat is the most important factor determining whether a population of *H. mantegazzianum* has persisted in a site or not, with meadow sites associated with a significantly higher probability of persistence than other sites. Additional factors such as urbanity, distance to origin, metapopulation connectivity and nearest-neighbour distance describe additional variation in the overall pattern.

The persistence of populations is still, after more than a century of ongoing invasion, significantly affected by proximity to the point of introduction (in 1877) and subsequent spread eastwards (mid-20th century) (Pyšek, 1991; Pyšek & Prach, 1993). This region is still highly invaded by *H. mantegazzianum* (Müllerová*et al.*, 2005), and our findings indicate that the resulting propagule pressure affects the long-term survival of populations up to *c.* 40–70 km from the focus of the original invasion.

However, the classification tree revealed one result that is somewhat counterintuitive in terms of the direction of the effect. In meadow sites, populations of *H. mantegazzianum* persisted with a higher probability if the metapopulation connectivity was low; that is, the more populations that were located within the 10-km perimeter, the lower the persistence. A possible explanation is that densely invaded areas may attract more investment in eradication, resulting in the successful removal of some populations. This conclusion seems to be partly supported by how the distance to the nearest neighbouring population affects the persistence of individual populations: among populations located outside settlements and far from the origin of invasion in the country, those located between 0.5 and 1.6 km from the next population showed the highest persistence rate. When above the upper threshold of this range, populations may be too far from each other to profit from the metapopulation connectivity, while those that are within the distance set by the lower threshold may suffer from the abovementioned tendency to attract eradication measures.

### Is our knowledge of the distribution of invasive species biased by the lack of data on their historical dynamics?

The knowledge of species distributions depends primarily on the scale of mapping, the quality of data, and the species itself (Mack, 2000; Hulme, 2003; Stohlgren, 2007). As precise actual distributions of species are rarely available, plant atlases usually rely on cumulative records gathered over the course of floristic surveys in a region (Petřík *et al.*, 2010). However, the persistence issue is largely neglected, even though it is highly relevant for invasive species with highly dynamic spread and an affinity for unstable human-made habitats (e.g. Chytrý*et al.*, 2005; Pyšek *et al.*, 2010).

The results of our study suggest that the use of cumulative records as a measure of species distribution, as is common in invasion literature, can result in serious overestimation of the current situation. In the case of *H. mantegazzianum*, the actual figure is approximately doubled (overestimated by 98%). Inferring the distribution of the species from cumulative historical data reported in the literature and herbaria suggests that 237 grid cells are occupied, while the estimate based on the knowledge of species persistence in invaded sites and systematic recent sampling gives 120 grid cells, a figure that is much closer to reality. The degree of bias associated with the distribution of invasive species is likely to be species-specific. However, the level of bias generally depends on the ability of the species to persist in invaded sites, with the inferred distribution becoming less and less realistic as the species’ persistence in the mapped region decreases. The deviation from reality of the inferred distribution based on historical data, which does not account for persistence, will therefore depend on modern floristic knowledge of the region as well as on the species persistence, which is related to the species traits and life history. In some dominant invasive species with clonal growth and high persistence in historical sites (up to 90%; Pyšek *et al.*, 2001), the distribution based on cumulative historical data is likely to be highly reliable.

There are other factors that affect the accuracy of the picture obtained by using past floristic records. First, the magnitude of the bias depends on scale – mapping individual sites will increase the bias, while using grid cells (132 km^2^ in size in our study) buffers the deviation if the species moves within the same grid. The bias at the level of grid cells can also be assumed to be lower if the species is widespread and abundant over most of the study region. The issue becomes even more relevant when working at a finer scale or with species that have a clustered distribution in the region of interest. If such overestimated distributions are used as a basis for studies of invasion dynamics, there may be an underestimation of real dispersal distances (Aikio *et al.*, 2010a) or, on the other hand, an overestimation of the invasion rate at larger scales (Fig. 3; Pyšek *et al.*, 2008). Another important factor is the stage of invasion: those invading species that have filled their potential ranges (on average around 150 years for neophytes in Europe, but this period can extend up to 400 years; Williamson *et al.*, 2009; Gassó*et al.*, 2010) are likely to be prone to less bias because they are not in the phase of dynamic spread.

*Heracleum mantegazzianum* is an example of an invasive species for which management measures act as confounding factors, limiting our capacity to predict its distribution based on ecological factors. It needs to be emphasized that the decisive role of management in shaping patterns of persistence in *H. mantegazzianum* limits the conclusions of our study to noxious invasive species that are often targeted for control or local eradication attempts (see Nielsen *et al.*, 2005 for an overview of its management in Europe). *Heracleum mantegazzianum* is also somewhat exceptional because it is easy to detect and because of its impact on human health (Pyšek, 1991; Müllerová*et al.*, 2005). While these particular traits of *H. mantegazzianum* may make our results less generally applicable to the majority of invasive plant species, they nonetheless show that overestimating the existing infested area is likely to impact negatively on choosing the optimal eradication strategy (Panetta *et al.*, 2011). As eradication success in plants depends on the total area infested (Rejmánek & Pitcairn, 2002), the correct determination of the target species distribution is a key aspect in deciding whether or not the eradication or containment is feasible.

## BIOSKETCH

**Jan Pergl** works at the Institute of Botany in the Academy of Sciences of the Czech Republic, and his research focuses on the population ecology of invasive plant species, by modelling their spread, and on macroecological patterns in biological invasions. He is actively involved in the management of and policy issues linked to invasive species in the Czech Republic.

The authors are members of a group studying various aspects of biological invasions in Europe. In recent years, they have been involved in European Union projects addressing biological invasions such as GIANT HOGWEED, ALARM, DAISIE and PRATIQUE.

Author contributions: J.P. and P.P. conceived the ideas; J.P. and I.P. collected the data; V.J. took the lead in data analysis; all authors contributed to writing the paper.
